# The role of silicon in enhancing resistance to bacterial blight of hydroponic- and soil-cultured rice

**DOI:** 10.1038/srep24640

**Published:** 2016-04-19

**Authors:** Alin Song, Gaofeng Xue, Peiyuan Cui, Fenliang Fan, Hongfang Liu, Chang Yin, Wanchun Sun, Yongchao Liang

**Affiliations:** 1 Key Laboratory of Plant Nutrition and Fertilizer, Ministry of Agriculture, Institute of Agricultural Resources and Regional Planning, Chinese Academy of Agricultural Sciences, Beijing 100081, P.R. China; 2 Ministry of Education Key Laboratory of Environment Remediation and Ecological Health, College of Environmental & Resource Sciences, Zhejiang University, Hangzhou, 310058, China

## Abstract

Here we report for the first time that bacterial blight of rice can be alleviated by silicon (Si) added. In both inoculated and uninoculated plants, shoot dry weight was significantly higher in the +Si plants than in the −Si plants. A soil-cultured trial showed that disease severity was 24.3% lower in the Si-amended plants than in the non-Si-amended plants. Plants that were switched from −Si to +Si nutrient solution and simultaneously inoculated with *Xoo* also exhibited the same high resistance to bacterial blight as the plants that were treated continuously with Si, with control efficiencies of 52.8 and 62.9%, respectively. Moreover, total concentrations of soluble phenolics and lignin in rice leaves were significantly higher in the +Si plants than in the −Si plants. Polyphenoloxidase (PPO) and phenylalanine ammonia-lyase (PAL) activities in rice leaves were observed to be higher in the +Si plants than in the −Si plants. The expression levels of *Os03g0109600, Prla*, *Rcht2* and *Lox2osPil*, were also higher in +Si plants than in −Si plants post-inoculation during the experimental time. Addition of Si resulted in increased *Pal* transcription, and inhibited *CatA* and *Os03g0126000* expression in the earlier and later stages of bacterial inoculation, respectively.

Bacterial blight caused by *Xanthomonas oryzae* pv. *oryzae* (*Xoo*) in rice (*Oryza sativa* L.) is one of the most destructive diseases restricting worldwide rice production. Since effective chemical control of this disease is lacking, breeding of rice varieties that are tolerant to bacterial blight is currently the only viable control measure. However, the resistance of cultivars with one or two major resistance genes is not stable under field condition because of the high pathogenic variability, and rapid evolution of virulent races. The breeding of rice cultivars with high levels of durable resistance would be ideal but little progress has been made in this regard. Therefore, it is necessary to develop alternative and novel technologies to control rice bacterial blight.

Plant-pathogen interactions are mediated by a complex network of molecular and cytological events that determine a range between susceptibility and resistance. Several defense-related enzymes are known to play a crucial role in the expression of host resistance. Phenylalanine ammonia-lyase (PAL) is considered to be the principal enzyme of the phenylpropanoid pathway, catalyzing the transformation of L-phenyalanine into trans-cinnamic acid, which in turn enters different biosynthetic pathways leading to the synthesis of lignin, a major product of phenylpropanoid metabolism. This defense mechanism is used for protection against pathogen invasion. The level of PAL activity is the key to the control of the synthesis of phenylpropanoids; PAL, therefore, has become one of the most extensively studied enzymes in plants. Induction of PAL has well been documented as a response to pathogen infection in various host-pathogen interactions^1^. The role of polyphenoloxidase (PPO) in disease resistance is to oxidize phenolic compounds to quinines, which are often more toxic to plant pathogens than the original phenols. They also play a significant role in lignin biosynthesis. The enzyme is localized in plastids, whereas its phenolic substrates are present mainly in the vacuoles^2^. PPO is implicated as having a key role in plant defenses against both insects and plant pathogens, and higher activities of PPO have been observed in resistant cultivars than in susceptible ones^3^. Chitinase is an enzyme that can hydrolyze the cell walls of pathogens, and it plays an important role in resisting pathogens, especially pathogenic fungi. Some studies suggested that chitinase activity was enhanced during the induction of resistance^4^,^5^. Lipoxygenase (LOX) can directly attack the plant membrane system in a non-enzymatic manner and activate the membrane’s lipid peroxidation. In addition, the metabolites can participate in the resistance response as an antibacterial or insect-resistant substance. Rusterucci *et al*.^6^ found a high LOX activity in cells around the dry spots in leaves, suggesting the LOX-dependent peroxide pathway resulted in the tissue necrosis of the hypersensitive response (HR). Previous studies showed that the accumulation of H_2_O_2_ was found in the thickened cells of the secondary cell wall near the infection point 5–8 h after infection. High concentrations of H_2_O_2_ accumulation in particular areas not only have a direct action against the microorganisms but can also cause fixed-point damage to cell membranes and accelerate the formation of dry spots^7^. Moreover, catalase (CAT) is an enzyme existing *in vivo* that is able to decompose H_2_O_2_ into water and molecular oxygen.

Applications of silicon (Si) fertilizers to rice can control rice blast^8^,^9^, sheath blight^10^ and brown spot^11^. Recently, we reported that Si could control bacterial blight (*Xoo*) in rice grown hydroponically^12^. The previous results also showed that Si could participate in the metabolic process of plant host pathogen interaction systems, activating the defense genes of hosts through a series of physiological and biochemical reactions and signal transduction, as well as inducing the resistance expression in plants, which is similar to the mechanism of resistance to fungal diseases in plants^13^,^14^. However, little information is available on the physiological role of Si in enhancing rice resistance to bacterial blight (*Xoo*), and especially, the molecular aspects. Furthermore, there is little information on Si and bacterial disease interactions in plants, in contrast to numerous reports on Si-enhanced resistance to fungal diseases. Therefore, this study aimed 1) to evaluate the role of Si in enhancing rice resistance to bacterial blight (*Xoo*) in both hydroponic- and pot-cultured rice; 2) to examine the influence of Si on phenolic compounds and their relevance to bacterial blight resistance; and 3) to investigate the induction and regulation of Si on defense-related genes.

## Results

### Dry weight and silicon concentration

**Figure 1:**
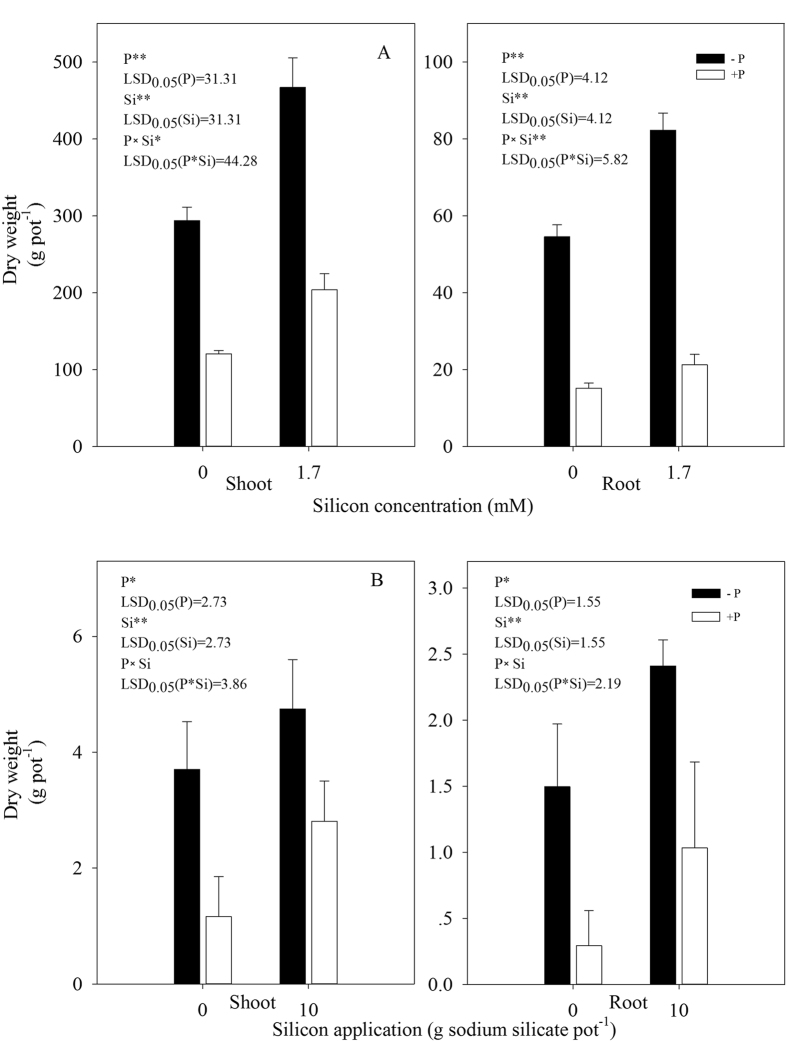
Dry weight of shoots and roots of rice seedlings non-inoculated (−P) or inoculated with *Xoo* (+P) under different silicon treatments in hydroponic (**A**) and soil-cultured (**B**) experiments. Bars are mean value of three replicates. Vertical lines represent standard deviations.

In both the hydroponic and the soil-cultured experiments, both root and shoot dry weights were significantly lower in the +P (*Xoo* inoculated) plants than in the −P plants (without inoculation) (Fig. 1A,B). Regardless of inoculation, shoot dry weight was significantly higher in the +Si (Si-amended) plants than in the −Si (non-Si-amended) plants.

**Figure 2:**
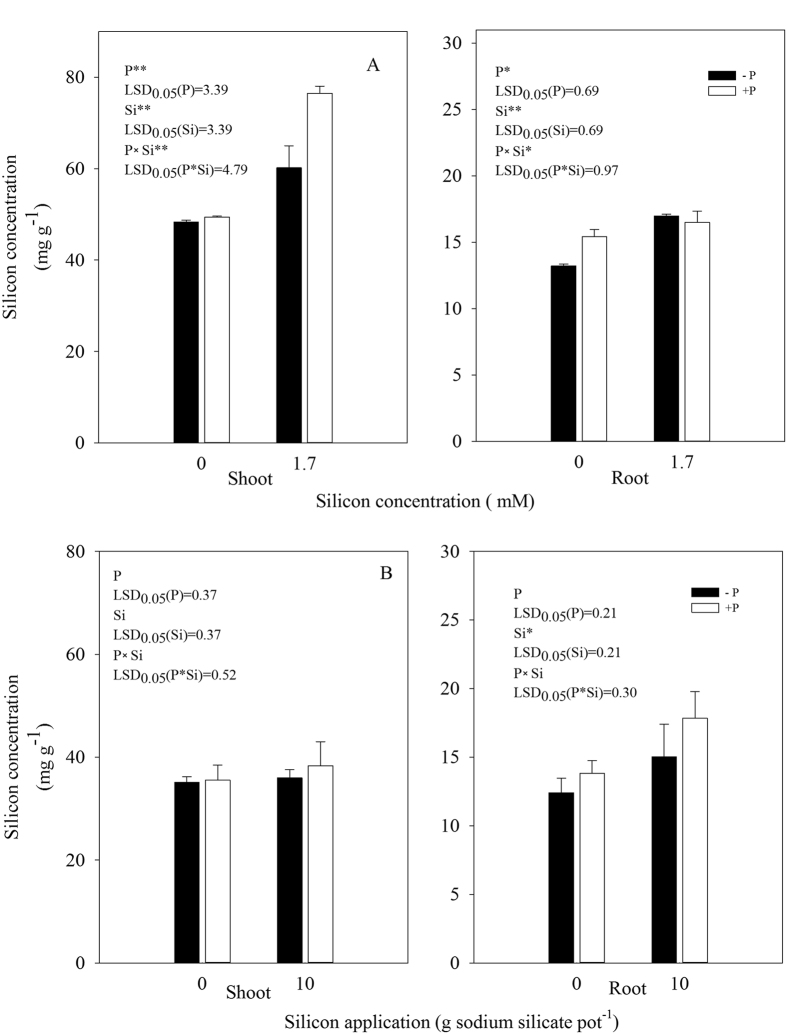
Silicon concentration of shoots and roots of rice seedlings non-inoculated (−P) or inoculated with *Xoo* (+P) under different silicon treatments in hydroponic (**A**) and soil-cultured (**B**) experiments. Bars are mean value of three replicates. Vertical lines represent standard deviations.

In the hydroponic experiment, both shoot and root Si concentrations were significantly higher in the +Si plants than in the −Si plants (Fig. 2A). The Si concentrations in all treatments were significantly higher in shoots than in roots. The shoot Si concentration was significantly higher in the +Si + P plants than in the +Si − P plants (Fig. 2A). However, there were no significant differences in Si levels between the −Si + P treatment and the −Si − P treatment. The root Si concentration was significantly higher in the −Si + P plants than in the −Si − P plants. However, there were no significant differences in root Si concentration between the +Si + P plants and the +Si − P plants.

For the soil-cultured experiment, no significant difference was found in the shoot Si concentration between the +Si + P treatment and the +Si − P treatment (Fig. 2B), No significant difference in Si levels was noted between the −Si + P treatment and −Si − P treatment, either. The root Si concentration was higher in the −Si + P plants than in the −Si − P plants, and similarly, it was higher in the +Si + P plants than in the +Si − P plants (Fig. 2B). Nevertheless, there were no significant differences in root Si concentration either between the −Si + P plants and the −Si − P plants or between the +Si + P plants and the +Si − P plants (Fig. 2B).

### Severity index

The leaf tissues in *Xoo*-inoculated sites quickly developed necrosis, and the disease spots stopped leaf elongation compared with the control plants. The symptoms of desiccation, immature death, leaf curlin and wilt were developed in leaves of the −Si plants. In the hydroponic experiment, the severity of disease development was significantly lower in the +Si plants than in the −Si plants with disease severity indices of 24.6%, and 66.1%, respectively (Table 1).

Rice plants initially grown in the −Si nutrient solution and switched into the +Si nutrient solution prior to inoculation (−Si+Si) also exhibited attenuated disease symptoms, with a disease severity index of 31.0% (Table 1). However, the level of resistance was 26% lower (significant at *P* < 0.05) in the −Si + Si plants than in the +Si plants (Table 1). The highest ratings for bacterial blight were 9, 5 and 5 for the −Si, −Si + Si, and +Si treatments, respectively (Table 1). Compared with the −Si treatment, the relative levels of rice bacterial blight in the −Si + Si and +Si treatments were 52.8% and 62.9%, respectively (Table 1).

In the soil culture experiment, the severity of disease development was 24.3% lower in the +Si plants than in the −Si plants, with disease severity indices of 72.0%, and 95.2%, respectively (Table 1). The control efficiency in the +Si treatment was 25% as compared with the control treatment (−Si). To our knowledge, this is the first trial indicating that Si amendments may reduce the impact of rice bacterial blight in the pot-cultured condition.

### The activities of PAL and PPO and the levels of soluble phenolics and lignin in rice leaves

**Figure 3:**
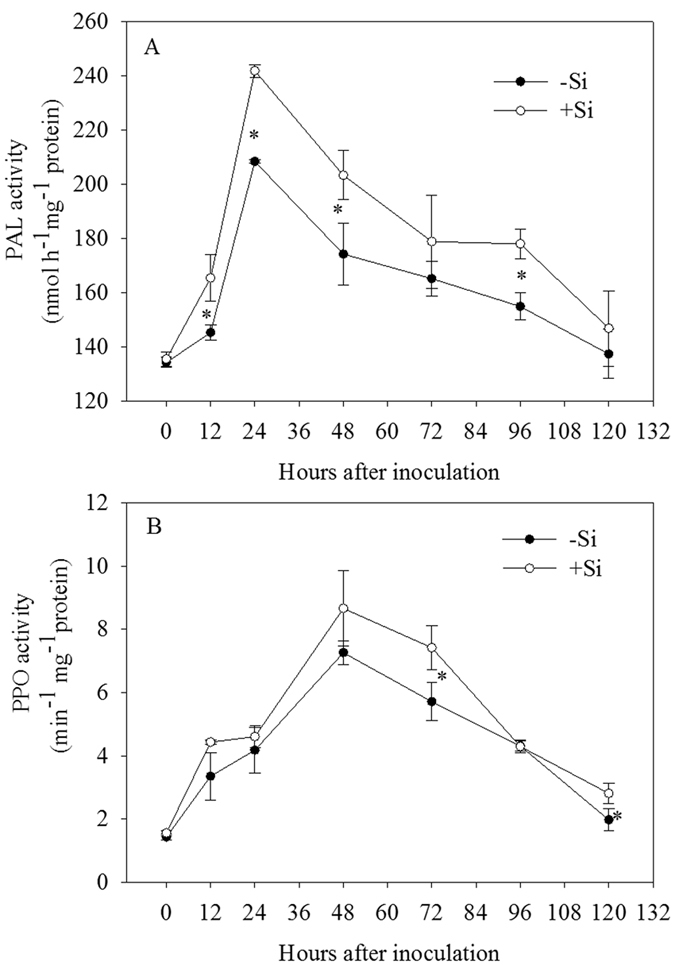
Changes in activities of phenylalanine ammonia-lyase (PAL) (**A**) and polyphenoloxidase (PPO) (**B**) in leaves of rice seedlings amended with silicon (+Si) or not (−Si) post-inoculation with *Xoo*. The values are means of three replications. Vertical lines represent standard deviations. Asterisks denote significant difference at *P* < 0.05 between +Si and −Si treatments at a same time-point according to Student’s *t*-test.

**Figure 4:**
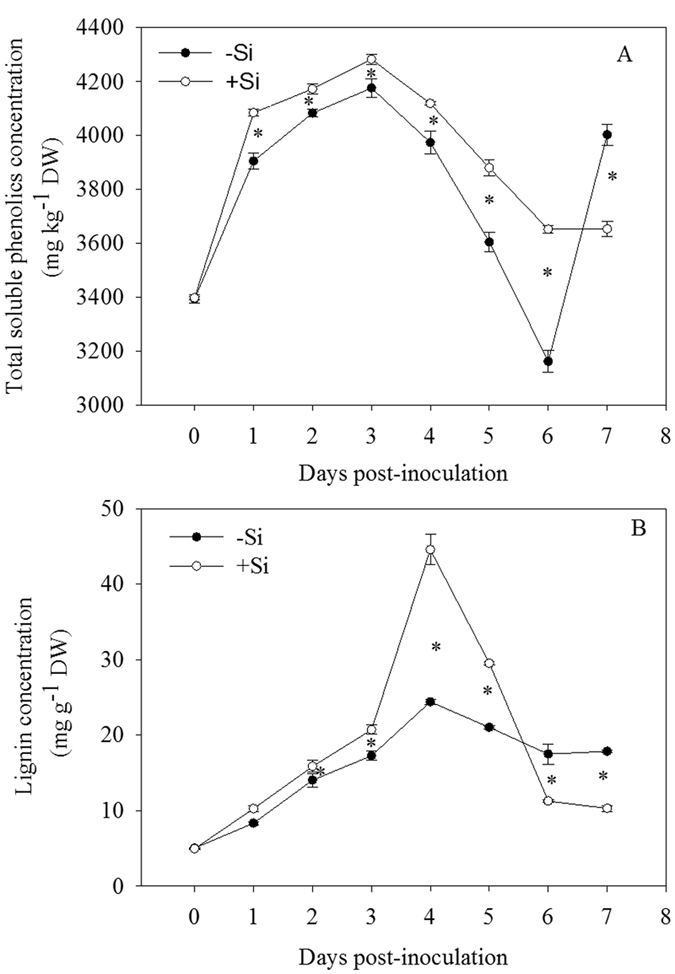
Changes in concentrations of total soluble phenolics (**A**) and lignin (**B**) in leaves of rice seedlings amended with silicon (+Si) or not (−Si) post-inoculation with *Xoo*. The values are means of three replications. Vertical lines represent standard deviations. Asterisks denote significant difference at *P* < 0.05 between +Si and −Si treatments at a same time-point according to Student’s *t*-test.

In the hydroponic experiment, PAL activity in leaves was significantly higher in the +Si plants than in the −Si plants throughout the experimental period, except at 72 h and 120 h post-inoculation (hpi). The PAL activity in both the +Si plants and the −Si plants reached a peak at 24 hpi (Fig. 3A). The enzyme activity then began to decline in both the +Si plants and the −Si plants. The PPO activity in rice leaves was higher in the +Si plants than in the −Si plants throughout the hydroponic experimental period, although this was significant only at 72 and 120 hpi (Fig. 3B). The PPO activity in both the +Si plants and the −Si plants reached a peak at 48 hpi (Fig. 3B). The level of total soluble phenolics in rice leaves was significantly higher in the +Si plants than in the −Si plants at 1–6 d post-inoculation (dpi) (Fig. 4A). The concentration of total soluble phenolics in both +Si and −Si plants reached a maximum at 3 dpi (Fig. 4A). The concentration of lignin in rice leaves was significantly higher in the +Si plants than in the −Si plants at 2, 3, 4 and 5 dpi (Fig. 4B). However, there was no significant difference between the +Si plants and the −Si plants at 1 dpi (Fig. 4B). The concentration of lignin in both the +Si plants and the −Si plants reached their maximum at 4 dpi (Fig. 4B).

### The expression levels of different genes in rice leaves

**Figure 5:**
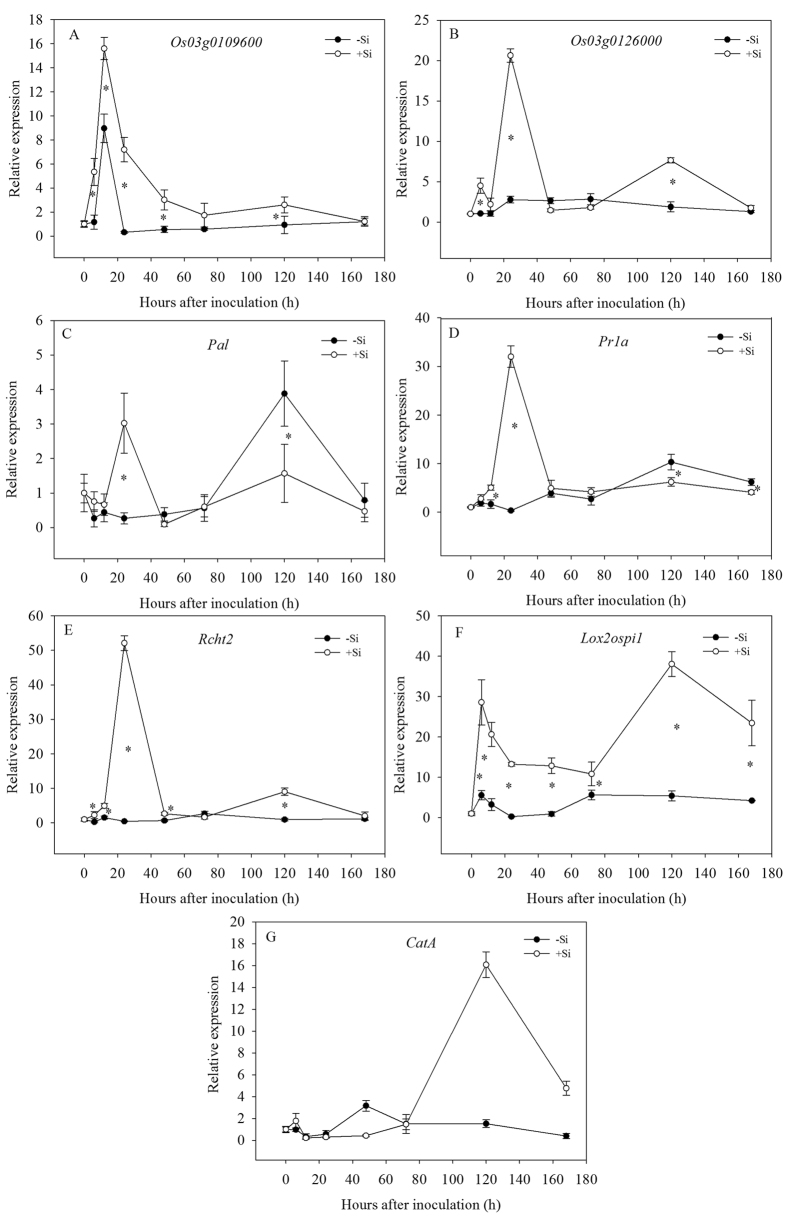
Relative expression levels of (**A**) *Os03g0109600*, (**B**) *Os03g0126000*, (**C**) *Pal*, (**D**) *Prla*, (**E**) *Rcht2*, (**F**) *Lox2osPil*, and (**G**) *CatA* genes in leaves of rice seedlings amended with silicon (+Si) or not (−Si) post-inoculation with *Xoo*. The values are means of three replications. Vertical lines represent standard deviations. Asterisks denote significant difference at *P* < 0.05 between +Si and −Si treatments at a same time-point according to Student’s t-test.

*Os03g0109600* gene encodes a protein which consists of 175 amino acids and contains nuclear localization signal and NAC conserved domain database. In the hydroponic experiment, there was a difference in *Os03g0109600* expression levels between the +Si plants and the −Si plants post inoculation. The expression levels at 6 and 24 hpi were 4.6 and 22.3 times higher, respectively, in the presence of Si than in the absence of Si (Fig. 5A). The *Os03g0109600* gene might play an important role in resistance to the bacterial blight of rice. The addition of Si could activate *Os03g0109600* gene expression and enhance the activity of transcription factors, consequently increasing disease resistance.

Rice binding protein *Os03g0126000* gene with 2-Hacid_dh_C conservative domain structure located in the chromosome 3, may encode for a protein with 424 amino acids, and may be the C-terminal binding protein. There was no significant change in *Os03g0126000* expression in the −Si plants throughout the hydroponics experimental period. However, the expression level increased more quickly in the presence of Si than in the absence of Si and reached a peak at 24 hpi, and the levels were 4.5 and 20.6 times higher, respectively, at 6 and 24 hpi than at 0 hpi (Fig. 5B). The expression levels were higher in the +Si plants than in the −Si plants before 24 hpi. For example, the RNA level of the *Os03g0126000* gene in plants treated with Si was 4.3 and 7.5 times as high as those in plants not treated with Si at 6 and 24 hpi, respectively (Fig. 5B). However, the expression level was higher in the Si-deprived plants than in the Si-treated plants at 48 and 72 hpi.

The relative expression level of the *Pal* gene was inhibited when there was no Si treatment during the hydroponics experimental period, except at 120 hpi (Fig. 5C). The expression level was also inhibited, except at 24 and 120 hpi with addition of Si. The RNA level for the *Pal* gene in the +Si plants was even higher than in the −Si plants. At 6 h and 24 hpi, the RNA levels for the *Pal* gene in the +Si plants were 2.8 and 11.3 times, respectively, as great as those in the −Si plants (Fig. 5C). In the early susceptible stage, the addition of Si could regulate *Pal* gene expression and enhance the resistance to the bacterial blight of rice.

The *Prla* gene is a PR-1 gene encoding a pathogenesis-related protein. As shown in Fig. 5D, its relative expression levels did not change significantly during the early stages of infection in the −Si plants, but were suppressed at 24 hpi in the hydroponic experiment. The expression levels rapidly increased and reached a maximum at 24 hpi in the +Si plants, which were 5 times and 32 times higher at 12 and 24 hpi, respectively, than at 0 hpi. The *Prla* gene’s expression level was significantly higher in the +Si plants than in the −Si plants before 72 hpi. The level was 3 and 97 times greater in the +Si plants than in the −Si plants at 12 and 24 hpi, respectively. These results showed that the addition of Si induced *Prla* gene expression earlier and faster, consequently enhancing resistance to the bacterial blight of rice.

The *Rcht2* gene is one of the genes encoding for chitinase which is a kind of proteins widely existing in microorganism and plants, and is a secondary hydrolytic enzyme related to defense in plants. As shown in Fig. 5E, the *Rcht2* gene’s expression level was significantly higher in the +Si plants than in the −Si plants during the hydroponics experimental period, except at 72 and 168 hpi (Fig. 5E). Moreover, the expression rate in the +Si plants was more rapid than in the −Si plants. The expression level was 7.9 and 114.3 times as high in the +Si plants as that in the −Si plants at 6 and 24 hpi (Fig. 5E). The results showed that the *Rcht2* gene might play an important role in resistance to rice bacterial blight. The addition of Si could activate the expression of the *Rcht2* gene, and enhance the resistance to bacterial blight.

*Lox2osPil* expression in the +Si plants was significantly higher than in the −Si plants at all time points in the hydroponic experiment. For example, the expression level was, respectively, 5.2 and 53.5 times greater in the +Si plants than in the −Si plants at 6 and 24 hpi (Fig. 5F). The expression level of the *Lox2osPil* gene rapidly increased in the +Si and −Si plants and was 5.6–28.6 times higher at 6 hpi than at 0 hpi (Fig. 5F). Interestingly, the expression level of *Lox2osPil* was inhibited at 24 and 48 hpi without the addition of Si, however, the expression level was up-regulated by Si added at 24 and 48 hpi compared with that at 0 hpi.

The relative expression levels of the *CatA* were not significantly changed at 6 hpi, with or without the addition of Si, and were significantly inhibited at 12 and 24 hpi in the hydroponic experiment. The *CatA* expression level was always lower in the +Si plants than in the −Si plants from 12 to 72 hpi, which was counteracted later (Fig. 5G). The results showed that Si could inhibit the expression of *CatA* gene during the early stages of inoculation when bacterial blight and rice were interacting.

## Discussion

The nutritional and physiological effect of Si on plants has been well demonstrated for years. For example, Si can enhance the growth of plant organs, and improve the mechanical properties and the resistance to abiotic or biotic stress^15^,^16^,^17^,^18^. In this study, the results showed that addition of Si could increase the dry weight of rice plants inoculated with the bacterial blight pathogen or not in both the hydroponic and the soil-cultured experiments (Fig. 1A,B). These results further showed the Si had a positive effect not only on rice growth, but on disease resistance to bacterial blight (Table 1).

Silicon is able to enhance plant resistance against fungal attack^8^,^9^,^15^,^19^. Less information, however, is available about Si effect on plant resistance to bacterial infection, except for the pioneering researches on the interactions of silicon and bacterial wilt in tomato, a silicon non-accumulating plant species^20^,^21^,^22^.

Winslow *et al*.^23^ found that there was a negative correlation between the severity of disease and Si concentration in organization. These silica opals are stored in the form of noncrystalline silica minerals deposited within cells and on cell walls of different plant organs when monosilicic acid, is taken up by plant roots and transported to the aboveground organs, thus forming a physical barrier of strong defense capability^24^,^25^. Those are effective in not only preventing fungal invasion, but also reducing the enzymes degradation secreted by the fungi in cell walls of rice. However, there are still arguments that Si participated in the mutual antagonism process between plant host and pathogen, and activated host defense genes in plants, induced disease resistance protein and resisted disease invasion after the signal transduction. In this study, shoot and root Si concentration was higher in the +Si + P plants than in the −Si + P plants (Fig. 2A,B). Moreover, disease index was significantly lower in the +Si + P plants than in the −Si + P plants in both the hydroponic- and soil-cultured experiments (Table 1), similar to the enhanced resistance to fungal blast of Si-treated rice plants, where the pathogen is a fungus (*Magnaporthe grisea* (T.T. Hebert) M.E. Barr)^9^. This phenomenon suggested that Si deposited on the cell walls, which is immobilized and unavailable for redistribution or physiological activity, still affects bacterial blight development of rice, possibly by physically strengthening the cell walls. One of the well-recognized mechanisms involved in silicon-mediated resistance to fungal diseases in plants is the role of silicon as a physical or mechanical barrier^8^,^9^,^19^. This is true for studies on experiments with blast in rice and powdery mildew in cucumber, where high concentrations of fungal conidia were sprayed onto the host as inoculum^9^,^15^. In these trials, the silica deposited on the leaf cell walls was able to restrict the penetration of fungal hypha into plant tissues, thus protecting plants from fungal attack. However, in the present study, rice plants were inoculated by the leaf clipping method. In this case, bacterial infection through wounded leaf tissues could be expected and restriction of bacterial penetration by silica deposited on the cell walls was not possible, suggesting that silicon-enhanced resistance to bacterial blight of rice is not a consequence of mechanical or physical barriers, but is due to active and physiological host resistance responses.

PAL participates in the synthesis of plant secondary antiviral substances, which can measure the ability of plant disease resistance and play an important role in plant disease resistance responses^26^. PPO, existing in the cytoplasm in free form or bounding to mitochondria, chloroplasts and other subcellular organelles, is the main enzyme of phenolic substances oxidation. Many studies show that there is a positive correlation between the activity of PPO and the plant disease resistance^24^. In addition, PPO is also involved in the synthesis of lignin, enhancing the antibacterial ability of host. Liang *et al*.^27^ found that the PPO activity of the third leaf with addition of Si after inoculation was increased in anthracnose-infected cucumber. In this study, PAL and PPO activities in rice leaves were higher in the +Si plants than in the −Si plants (Fig. 3), which helped to enhance the resistance to bacterial blight of rice. Moreover, phenols are production of the secondary metabolites and play an important role in the process of metabolism and disease resistance. In this study, the total concentrations of soluble phenolics were increased rapidly and reached the peak at 3 dpi, and then decreased with or without addition of Si (Fig. 4A). Addition of Si could significantly increase the total concentrations of soluble phenolics and enhanced the resistance to bacterial blight. In previous studies, a large amount of soluble phenolic compounds could be accumulated in plants, which played an important role in plant disease resistance through delaying the growth of invading pathogens^28^. Lignin is a polymer of phenolic compounds, which is synthesized through the pathway of phenylpropanoid. Many studies suggested that the accumulation of lignin in plants had a close relationship with plant disease resistance^29^,^30^. The results showed the lignin concentration was rapidly increased after inoculation of bacterial blight regardless of addition of Si (Fig. 4B). Furthermore, the concentration of lignin in rice leaves was significantly higher in the +Si plants than in the −Si plants at 2, 3, 4 and 5 dpi (Fig. 4B). These findings are in agreement with previous studies demonstrating that +Si wheat plants produced enhanced levels of phenolic materials and phytoalexins in response to powdery mildew infection^31^,^32^, suggesting that Si might boost biochemical defense reactions in rice leaves by accelerating the biosynthesis of phenolics, phytoalexin and lignin. The presence of phenolic compounds in plants and their synthesis in response to infection are associated with resistance^33^. It has also been reported that accumulation of higher concentrations of phenolics due to increased PAL activity is associated with the expression of disease resistance by plants. This is associated with the release of antimicrobial compounds, and deposition of lignin to change the architecture of host cell walls, to provide barriers to restrict pathogen ingress, as found in many host-pathogen interactions^1^. Silicic acid can interact with pectins and polyphenols in the cell walls, and is mainly located in the cell walls^34^. Some of the silicon bound to cell walls is likely present as an ester-like derivative of silicic acid, which acts as a bridge in the structural organization of polyuronides, and affects the content and metabolism of polyphenols in xylem cell walls^34^. It seems that silicon may affect the stability of higher plants not only as an inert deposition in lignified cell walls but also by modulating lignin biosynthesis to mediate resistance to fungal or bacterial attack.

Plants developed a complex defense reaction system that was antagonistic to pathogen infection during the co-evolution of plants and pathogenic microorganisms. The plant defense response genes are induced by elicitor (specific and non-specific) induction, and cause defense-related plant disease resistance genes to be expressed. Their translation products play a direct inhibitory effect on pathogen proliferation. The defense reaction system was divided into four categories, i.e. secondary material synthesis genes, hydrolase and pathogenesis-related genes, genes related to cell wall modification and cell defense enzyme system related to removing reactive oxygen.

To investigate the beneficial effects of Si on plant disease resistance inoculated with *Xoo*, we examined the expression of rice genes in response to *Xoo* and Si treatments by real time RT-PCR. The expressions of many defense genes are induced when plants are suffering from pathogen infection. The gene encoding PAL is an important defense gene. Plants deposit many materials, including phytoalexin, lignin and other phenolic compounds on the cell walls by proteins (such as chitinase and, pathogenesis-related protein), which are synthesized by the phenylaprapanoid metabolism pathway. PAL is a key enzyme in this pathway, which provides a precursor for the synthesis of lignin and phytoalexin^35^. Some studies have shown that PAL plays an important role in the early reactions of plant defense.

An increase in the PAL enzyme level appeared in parsley leaf tissue experiencing allergic necrosis^36^. The transcript levels of *Pal* reached the highest level 16 h after inoculation of brown rust in oat and then began to degrade^37^. The HR could also be inhibited if PAL was suppressed in barley. Those results indicated that the product of the *Pal* gene is important for plant resistance to disease. The *Lox2osPil* gene in many plants is induced by injury and pathogens, such as blast fungus and bacterial blight in rice^38^. The enzyme activity of LOX increases during infection^39^. The tobacco parasitic phytophthora could induce *Lox2osPil* gene expression, resulting in hypersensitive cell death^40^. The sensitivity to tobacco diseases and root rot was increased when the antisense DNA of the *Lox2osPil* gene was transferred into tobacco^41^.

*Os03g0109600*, containing six exons and five introns, is mainly located in the nucleus and the cell membrane and functions as a transcriptional repressor. It is reported to be regulated by bacterial blight of rice^42^. In the present study, the *Os03g0109600* gene expression was activated in *Xoo*-infected plants by addition of Si, which was higher than in the Si-untreated plants. Si could enhance transcription factor activity and improve disease resistance (Fig. 5A). Later in the infection process, the expression levels of *Os03g0126000* gene were inhibited with the addition of Si (Fig. 5B). The protein encoded by the *Os03g0126000* gene was a transcriptional repressor, which helped to maintain the normal physiological metabolism and disease resistance response.

The expression level of *Pal* was regulated by Si after *Xoo* inoculation. At the beginning of the disease, the expression level of *Pal* was higher with addition of Si than without addition of Si, which was contributed to increased activity of PAL (Fig. 5C). As is well known, PAL is the key enzyme in the process of phenolic substances metabolism. Si could induce *Prla* and *Rcht2* genes to express earlier and faster, as well as produce pathogenesis-related proteins faster, which would have an inhibitory effect on the pathogen (Fig. 5D,E).

The addition of Si could significantly increase the *Lox2osPil* gene expression levels and regulate *CatA* gene expression during the interaction of rice and bacterial blight (Fig. 5F,G). *CatA* gene expression was significantly inhibited, which was helpful for HR and enhanced the resistance to bacterial blight of rice. Taken together, these results suggest that a complex physiological and biochemical process resulting from the induction of defense genes by the addition of Si is the main mechanism of the resistance to bacterial blight. This is the first study using real-time PCR to quantitatively assess the effect of Si on regulation of defense genes in rice infected by bacterial blight^12^,^43^.

## Conclusions

Si fertilization increased the resistance of rice plants to *Xoo* in both hydroponic and soil-cultured conditions. The increased uptake of Si led to increased synthesis of total soluble phenolics and lignin, activities of PAL and PPO, and regulation of defense genes including *Prla*, *Rcht2, Pal*, *Lox2osPil, CatA, Os03g0109600* and *Os03g0126000*. Silicic acid played an active physiological role in priming and enhancing resistance to bacterial blight of rice. Therefore, application of Si fertilizers might be an effective option for enhanced resistance to bacterial blight of rice.

## Methods

### Experimental design and sampling

#### Experiment A

A 2 × 2 factorial hydroponic experiment, consisting of rice plants amended with silicon (+Si) or not (−Si) and inoculated with *Xoo* (+P) or not (−P), was arranged in a completely randomized design with three replicates per treatment. There were 30 plant seedlings per pot. The shoots and roots of 30 seedlings per pot were separately harvested at 20 days post-inoculation (dpi). The shoots and roots were washed thoroughly with double-distilled water and dried in an oven at 70 °C for 3 days until a constant weight was reached, then weighed for determination of dry weight, and subsequently ground with an ultra centrifugal mill (Retsch Gmbh, Germany) to a powder (0.5 mm sieve) for analysis of silicon.

#### Experiment B

A 2 × 2 factorial soil-cultured experiment, consisting of rice plants amended with silicon (+Si) or not (−Si) and inoculated with (+P) or without *Xoo* (−P), was arranged in a completely randomized design with each treatment replicated three times. There were 5 plant seedlings per plastic pot. The shoots and roots of 5 rice seedlings per pot were separately harvested at 30 dpi and then pretreated in the same manner as described above for determination of dry weight and analysis of silicon.

#### Experiment C

Rice plants were grown in the nutrient solution amended with (+Si) or without (−Si) soluble silicon. Initially, the −Si treatment was replicated for 6 times and the +Si treatment for 3 times. At the onset of the fifth leaf stage, an additional treatment (−Si + Si) was established by switching half of the −Si treatments (3 pots) to the +Si treatments. The plants grown in the +Si, −Si and −Si + Si nutrient solutions were all immediately inoculated with *Xoo* (10^9^ cfu ml^−1^) after renewal of nutrient solution. Disease development was evaluated at 20 dpi.

#### Experiment D

Rice plants were grown in the nutrient solution amended without (−Si) or with 1.7 mM Si (+Si). Plants were subjected to inoculation with (+P) or without *Xoo* (−P). Each treatment was replicated 3 times. At 0, 1, 2, 3, 4, 5, 6 and 7 dpi, the third and fourth leaves from the main tiller of each plant were collected. Portions of the samples were used directly for analysis of PAL and PPO activities, and the remaining samples were freezing dried for 72 h, and ground together into a fine powder in a mortar and pestle under liquid nitrogen and then used for assay of total soluble phenolics and lignin.

#### Experiment E

Rice plants were grown in the nutrient solution amended without (−Si) or with 1.7 mM Si (+Si). Plants were subjected to inoculation with (+P) or without *Xoo* (−P). Each treatment was replicated 3 times. At 0, 6, 12, 24, 48, 72, 120 and 168 h post inoculation, the third and fourth leaves from the main tiller of each plant were collected for analysis of the gene expression by quantitative RT-PCR.

### Rice culture and silicon treatment

Plants were grown in a growth chamber under controlled conditions (photoperiod 14 h light/8 h dark; temperature 25/20 °C day/night; humidity 70%; light intensity 400 μmol m^−2^ s^−1^). Rice (*Oryza sativa* L. cv. Nipponbare), which is susceptible to *Xoo*, was used for studies of silicon-enhanced resistance to *Xoo* in this study. Rice seeds were surface sterilized with 0.5% (v/v) NaOCl for 15 min, rinsed thoroughly with distilled water, and soaked in distilled water for 24 h at 30 °C in the dark. The seeds were then transferred to a net floated on double-distilled water. After 2 weeks, uniform-sized seedlings were transplanted into 8-liter plastic containers with full-strength Kimura B nutrient solution^43^. The nutrient solution was prepared using purified water (Milli-Q Synthesis A10, Millipore Corporation, USA) and renewed every 4 days. The initial solution pH was adjusted to 5.6. Soluble silicon was fed to the plants by amending the nutrient solution with 1.7 mM Si in the form of sodium metasilicate nonahydrate (Na_2_SiO_3_.9H_2_O), which was neutralized with HCl prior to addition^44^. Control plants were continuously fed with full-strength Kimura B nutrient solution without silicon added.

For the soil-cultured experiment, the soil used was an anthropogenic soil, collected from Qinghuandao, Hebei province, China. The soil’s physico-chemical properties were: pH 5.96, organic matter 30.2 g kg^−1^, alkaline hydrolytic N 49.95, Olsen-P 18.86 mg kg^−1^, available K 136.76 mg kg^−1^, and available SiO_2_ (extracted by pH 4.0 acetate buffer) 231.08 mg.kg^−1^. The soil was air-dried, crushed to pass through a 2 mm sieve and mixed well with 0.25 g kg^−1^ N (urea), 0.15 g kg^−1^ P (KH_2_PO_4_), and 0.25 g kg^−1^ K (K_2_SO_4_ and KH_2_PO_4_). The pretreated soil was loaded into plastic pots (4 kg per pot) and kept flooded until the end of the experiment. Two treatments with three replicates each were established: i.e. −Si (control, with no Si added) and +Si (with Si added at 10 g sodium silicate per pot). Rice seeds were sterilized in the way described above in the hydroponic experiment and then soaked in distilled water at 30 °C in the dark for 3 days to germinate. After germinating, 8 seeds were sown into each plastic pot; finally each pot was thinned to 5 seedlings 10 days after sowing. Plants were grown in a growth chamber under controlled environmental conditions with light and temperature regimes described above, and were harvested 8 weeks after rice transplanting.

### Inoculation method

A *Xoo* strain, JXOI, was used throughout this study. For inoculum preparation, the bacterium was grown on NA (nutrient agar) medium at 28 °C for 48 h and then shaking culture in NB (nutrient broth) medium (150 r min^−1^) at 28 °C for 24 h. The inoculum was prepared by diluting the bacterial culture in sterilized water to a concentration of approximately 10^9^ colony forming units (cfu) per ml. At the onset of the fifth leaf stage, three to four of the uppermost fully expanded leaves of each plant were inoculated by the leaf clipping method^45^, in which the leaf blade was cut with a pair of scissors pre-dipped in the bacterial suspension. The control plants were inoculated with sterilized water instead. After inoculation, plants were transferred into a growth chamber. The relative humidity inside the growth chamber was maintained over 85% by using an atomizer (Defensor 3001, Axair Ltd., China) for the duration of the experiment.

### Determination of silicon concentration

The plant samples (around 0.2 g) were microwave-digested in a mixture of 3 ml of HNO_3_ (62%), 3 ml of H_2_O_2_ (30%), and 2 ml of HF (46%). Silicon concentration in the digested solution was determined by the colorimetric molybdenum blue method^44^.

### Disease evaluation

For disease scoring, lesion development on individual leaves at 20 dpi was rated according to the method of Fang *et al*.^46^, in which a rating of 0 means the absolute disease lesion length less than 0.2 cm; 1, 0.2–1.5 cm; 3, 1.5–3 cm; 5, 3–5 cm; 7, 5–10 cm; 9, over 10 cm. A disease index (%) was calculated using the following formula:
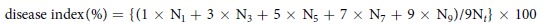
where N_1~9_ is the number of 1~9 Grade index leaves, and N_t_ is total number of leaves tested.

### Assays of PAL and PPO activity in rice leaves

PAL activity was measured colorimetrically at 290 nm following the method of Qin and Tian^47^, and was defined as nanomole cinnamic acid produced per hour per milligram of protein. The protein content of the enzyme extracts was estimated by the method of Bradford^48^ with bovine serum albumin (BSA) as the standard. PPO activity was also measured colorimetrically at 398 nm (A_398_) according to the method of Qin and Tian^47^, and expressed as A_398_ per minute per milligram of protein.

### Determination of total soluble phenolics and lignin concentrations in rice leaves

The total soluble phenolics concentration was determined colorimetrically at 725 nm and expressed as mg of phenolics (in terms of catechol) per kg of dried leaf tissue following the method of Rodrigues *et al*.^49^. The concentration of lignin and lignin-like phenolic polymers was determined colorimetrically at 280 nm according to the method of Rodrigues *et al*.^49^.

### Quantitative real-time PCR analysis

At 0, 6, 12, 24, 48, 72, 120 and 168 h post inoculation, plants were harvested and samples were collected to analyze the expression of genes by fluorescent quantitative real-time reverse transcription-polymerase chain reaction (qRT-PCR).

According to the testing results of pathogensis-related enzymes, five main genes encoding chitinase, lipoxygenase (LOX), phenylalanine ammonia-lyase (PAL), PR-1 pathogenesis-related proteins and catalase (CAT) were selected and further confirmed by quantitative real-time PCR. In addition, transcription gene *Os03g0109600* and binding protein gene *Os03g0126000* regulated by bacterial blight were also chosen.

Gene specific primers (Table 2) were designed and synthesized by Baosheng Corporation, China. Total RNA isolated from rice tissues was first converted to cDNA using MMLV reverse transcriptase (Tiangen Corporation, China), according to the manufacturer’s specifications, with random hexamer primers from Baosheng Corporation. The cDNA products generated were from three biological replicates for each sample. Reactions (20 μl) were performed in triplicate in 96-well plates (TempPlate Scientific, BIO-RAD, China). The program consisted of a Hot-Start activation step at 95 °C for 14 s, followed by 40 cycles of: 95 °C for 15 s, 58 °C for 30 s, 72 °C for 30 s. Amplification of the actin internal control was performed in the same 96-well plate as the other genes. Reactions were performed with the iCycler Real-Time PCR Detection System (Bio-Rad Laboratories INC., USA) employing the two-step amplification plus melting curve protocol. The values for threshold cycle (Ct) determination were generated automatically by iCycler software (Bio-Rad Laboratories). Relative quantitative method delta-delta C_T_ (2^−△△CT^ )^50^ was used to describe expression patterns of selected genes by comparing the gene expression levels between inoculation *Xoo* samples with or without Si and healthy samples.

### Statistical analysis

All experimental data reported were the means ± standard deviation (SD) of three replicates. The data in Figs 1 and 2 were examined statistically by analysis of variance (ANOVA). Statistical significances of the means were determined by Duncan’s new multiple range test at *P* < 0.05, using SPSS 13.0 for Windows (Chicago, IL, USA). The data in Figs 3, 4, 5 were examined statistically by Student’s-*t* test. Asterisks in Figs 3, 4, 5 denote significant difference at *P* < 0.05 between +Si and −Si treatments at a same time-point.

## Additional Information

**How to cite this article**: Song, A. *et al*. The role of silicon in enhancing resistance to bacterial blight of hydroponic- and soil-cultured rice. *Sci. Rep.* **6**, 24640; 10.1038/srep24640 (2016).

## Figures and Tables

**Table 1:** Effects of silicon fertilization on bacterial blight development in rice leaves in hydroponic and soil-cultured experiments.

Treatments	Highest disease level	Severity index	Efficiency (%)
Hydroponic experiment			
−Si	9	66.1 ± 5.7a	–
−Si + Si	5	31.0 ± 1.1c	52.8 ± 5.2
+Si	5	24.6 ± 4.4d	62.9 ± 4.8
Soil-cultured experiment			
−Si	9	95.19 ± 3.26a	–
+Si	9	72.04 ± 9.32b	25.0 ± 8.7

Data are means + SD of three replicates.

Note: The different letters in the same column indicate significance at the 0.05 level.

**Table 2:** The conserved region of gene sequence and predicted product size for PCR used for detecting gene expression in the leaves of rice.

Gene Name	Accession No	Primer sequence (5′-3′)	PCR product size (bp)
*Pal*	EF576408	F: 5′ -TTCCCGCTCTACCGCTTCGT -3′	163
		R: 5′ -GCTCGCCGTTCCACTCCTTG -3′	
*CatA*	EF371902	F:5′-CGTCATCGTCCGCTTCTCCACCGTC-3′	126
		R:5′-AAGTTGTTGCCGAGGAGGTCCCAGT-3′	
*Rcht2* (chitinase)	AB016497	F: 5′-AGATAAACAAGGCGACTTCTCCAC-3′	179
		R: 5′-CGCCGTCATCCAGAACCAG-3′	
*Pr1a*	EF061246	F: 5′-TCGGCGTGGGTGTCGGAG-3′	176
		R: 5′-GAGTAGTTGCAGGTGATGAAG-3′	
*Lox2osPil* (Lipoxygenase)	D14000	F: 5′-ACG TGC TGT CCA GCC ACT C-3′	133
		R: 5′-ATG ACG CCC TCG ATC TCC T-3′	
*Os03g0109600*	NM_001055244	F: CTGCTTCCAACAATCATCAACC-3′	133
		R: CGTCGTCGTCCTGAGCGTTA-3′	
*Os03g0126000*	NM_001055357	F: GAAATTAGCGAAGCTGGAAATG-3′	150
		R: TTGAGGCTCTTTATGCTGGGAT-3′	
*Actin*	AB047313	F: 5′-ACCCAAAGGCTAACAGAGAG-3′	173
		R: 5′-ACACCATCACCAGAATCAAG-3′	
